# Thermoplastic elastomers based on recycled high-density polyethylene/ground tire rubber/ethylene vinyl acetate: Effect of ground tire rubber regeneration on morphological and mechanical properties

**DOI:** 10.1177/08927057221095388

**Published:** 2022-04-25

**Authors:** Ali Fazli, Denis Rodrigue

**Affiliations:** 1 Department of Chemical Engineering, 4440 Université Laval, Quebec, QC, Canada

**Keywords:** Recycling, rubber regeneration, high-density polyethylene, ground tire rubber, compatibilization

## Abstract

This work investigates the properties of different types of regenerated recycled rubbers (RR_1_ and RR_2_) to produce thermoplastic elastomers (TPE) based on recycled high-density polyethylene (RHD) as the matrix. The higher regeneration degree of RR_2_ (24%) compared to RR_1_ (15%) was able to better restore the plasticity and processability of the ground tire rubber (GTR). So better entanglement between RR_2_ free chains and the thermoplastic macromolecules was obtained inducing stronger interfacial interaction leading to higher elongation at break (159%) and impact strength (342 J/m) of the blends filled with 80 wt.% RR_2_. To further improve the adhesion and achieve rubber-like properties, ethylene vinyl acetate (EVA) was used as a compatibilizer. The microstructure analysis showed that uniform dispersion of the particles and ground tire rubber encapsulation by EVA increased the resistance to crack propagation and failure of the compatibilized blends. The swelling, mechanical and physical properties of the ternary blends (RHD/GTR/EVA) showed that EVA improved the interfacial interactions between GTR and RHD which was confirmed by enhanced elongation at break (203%) and impact strength (379 J/m) by the addition of 10 wt.% EVA.

## Introduction

The reuse, recycling and recovery of waste tires, as one of the largest and most problematic waste materials, is an intensively studied topic to find new applications for ground tire rubber (GTR). Tires are mainly composed of vulcanized rubbers (crosslinked thermoset structure) and different additives (stabilizers, anti-oxidants, anti-ozonants, etc.) making such waste not degradable (very slow) and not reprocessable by direct melting like thermoplastics.^1,2^ Recently, the development and growth of thermoplastic elastomers (TPE) has gained significant attraction for using waste tires by producing polymer blends combining the elastomer properties of rubbers with the easy processability of thermoplastics.^3,4^ Several efforts have been made regarding the preparation and characterization of TPE containing GTR and thermoplastics such as low density polyethylene (LDPE),^5–7^ high-density polyethylene (HDPE),^8,9^ or polypropylene (PP).^10–12^ But most of the research focused on melt blending GTR with virgin thermoplastics, while TPE production based on recycled resins is more sustainable and environmentally friendly to produce green and inexpensive TPE materials.

HDPE is one of the most common polyolefins with applications in several markets (packaging, automotive, electrical, pipes and fittings) because of its good mechanical properties, excellent processability and low cost. Therefore, HDPE from recycled sources could produce a great advantage for GTR recycling.^13–15^ But TPE materials, based on a physical mixture of thermoplastic and GTR (thermoset material), are generally incompatible blends with low mechanical properties.^13^ It is well established that adding rubber with a crosslinked network leads to low tensile properties of the resulting thermoplastic blends which is attributed to incompatibility and poor interfacial interaction between the phases.^16^ Crosslinked rubber molecules, with restricted chain mobility, do not entangle with the matrix macromolecules to create suitable interaction leading to GTR particles agglomeration and voids formation around the rubber particles (poor interfacial adhesion and compatibility) resulting in low mechanical properties.^4^ But the addition of GTR with a vulcanized structure into a thermoplastic matrix increases the blend viscosity (processing torque) which results in difficult processing, especially as GTR content increases.^12,15^ In general, increasing the GTR concentration with a three dimensional (3D) crosslinked structure and restricted chain mobility decreases the tensile properties of the resulting blends, especially the elongation at break due to poor compatibility between the components. For example, Kakroodi and Rodrigue^13^ reported that the incorporation of GTR inside HDPE led to very low homogeneity as the elongation at break of GTR/HDPE (70/30) was only 64%, while further GTR addition significantly decreased this value down to 44% at a GTR/HDPE (90/10) ratio.

But TPE compatibility can be improved via different approaches: decreasing interfacial tension, morphology stabilization, size reduction of the dispersed phase and increasing the interfacial adhesion for better stress transfer. Several methods, such as partial regeneration^17,18^ and coupling agent addition,^16,19,20^ have been proposed to improve the compatibility between GTR and thermoplastics. Rubber crosslinked network breakdown through GTR regeneration can improve the rubber chain mobility (molecular freedom) and plasticity of recycled rubbers to promote molecular interactions and chains bonding through partially soluble fraction of regenerated rubber (RR).^21^ Also, GTR regeneration is able to induce lower friction between RR particles and lower viscosity of the blend leading to better processability.^22^ A comparison between the tensile properties of natural rubber compounds filled with 10 wt.% GTR or RR showed that blends filled with RR particles had higher tensile strength (23.2 MPa) and elongation at break (612%) compared to the tensile strength (13.7 MPa) and elongation at break (417%) of GTR based blends attributed to the presence of less carbon black and lower gel fraction (acting as stress concentration points) in RR blends.^23^ Shaker and Rodrigue^5^ reported that RR have smaller particle size and smoother surface compared to GTR due to particle break-up by shear/elongational forces associated with the thermomechanical regeneration process. It is expected that smaller RR particle size have higher specific surface areas compared to GTR particles resulting in improved filler distribution, stronger interfacial bonding and better stress transfer between the components.^5^

It is important to determine the relationships between the phase morphology and the mechanical properties of TPE. In general, a minimum of 100% elongation at break is required.^24^ This can be easily achieved with polyolefins (mainly polyethylene). But these resins are non-polar and have low affinity for blending with GTR or RR. Nevertheless, the addition of copolymers, such as ethylene vinyl acetate (EVA),^7,25^ polyolefin elastomer (POE),^20,26^ and styrene–butadiene–styrene block copolymer (SBS)^27^ can act as a bridge (coupling agent) to improve the interactions between the matrix and the rubber particles. These copolymers are practical compatibilizers to improve the interfacial adhesion and uniform distribution through the encapsulation of filler particles during melt mixing.^25,26^ EVA copolymers having rubber-like properties with excellent ozone, weather and stress-crack resistance are good compatibilizers for TPE.^28^ Mészáros et al.^25^ reported that EVA (20 wt.%) produced strong adhesion between LDPE and GTR as the elongation at break of LDPE/GTR blends increased by 60% (from 100% to 160%), while the rubber-like nature of EVA decreased the tensile modulus by 25% (from 80 MPa to 60 MPa). In a similar study, the addition of EVA as a polar compatibilizer into recycled LDPE (rLDPE)/LDPE/GTR (40/30/30) blends increased the elongation at break from 125% to 225% due to GTR encapsulation by EVA reducing the surface energy of the components to form a strong interface.^7^

In our previous work, recycled HDPE(RHD)/GTR blends (RR and non-regenerated (NR) recycled rubber contents between 0 to 90 wt.%) showed clear incompatibility and low interfacial adhesion between RHD and GTR (RR and NR) for all formulations. The results showed that melt blending of RHD with 80 wt.% of GTR yielded elongation at break of 129% and 50% for NR and RR blends, respectively. In this study, the main objective is to study the effect of GTR regeneration (evaluation of the regeneration degree and crosslink density) and blend composition on the swelling, morphological, mechanical and physical properties of highly filled TPE blends (above 70 wt.% GTR) by comparing different regenerated rubbers. NR and two types of RR particles (RR_1_ and RR_2_) in the range of 70, 80 and 90 wt.% were introduced into RHD via continuous melt-mixing in a twin-screw extruder and the specimens were compression molded for further analysis. Also, the effect of EVA content (5–15 wt.%) as a compatibility/interfacial adhesion promoter is investigated to produce ternary blends of RHD/GTR/EVA.

## Experimental

### Materials

The recycled high-density polyethylene (RHD) was kindly provided by Gaudreau (Victoriaville, Canada) and used as the matrix. This polymer has a melt flow index (MFI) of 1.3 g/10 min (190°C and 2.16 kg) according to ASTM D1238. The density (ASTM D2856) and melting point (ASTM D3418) of the RHD are 0.967 g/cm^3^ and 129.5°C, respectively. The ground tire rubber (GTR) particles were provided by Phoenix Innovation Technology (Montreal, Canada) from the same source of off-the-road (OTR) waste tires composed of natural rubber as the main component. Such GTR particles were exposed to two different regeneration process by Phoenix Innovation Technology and were used as received in three forms^29^: regenerated rubber (RR_1_) with an average particle size of ∼600 μm and a density of 1.246 g/cm^3^; regenerated rubber (RR_2_) with an average particle size of ∼500 μm and a density of 1.193 g/cm^3^; and a non-regenerated rubber (NR) with an average particle size of ∼300 μm and a density of 1.169 g/cm^3^. EVA as a recycled copolymer containing 25% of vinyl acetate, was kindly provided by Ecofib (Drummondville, Canada) and was used as received. This copolymer has a density of 0.946 g/cm^3^ and a MFI of 1.81 g/10 min (190°C and 2.16 kg) according to ASTM D1238.

### Processing

**Figure 1.**
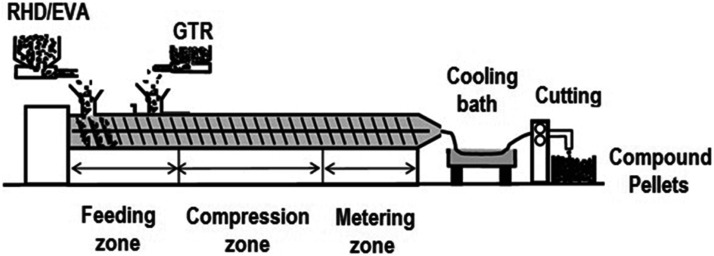
Schematic representation of the melt blending process for recycled high-density polyethylene/ground tire rubber/ethylene vinyl acetate compounds.

The RHD thermoplastic matrix, GTR (NR, RR_1_ and RR_2_) particles, and EVA were compounded using a Leistritz ZSE-27 twin-screw extruder with a L/D ratio of 40 and 10 heating zones coupled to a circular die (2.7 mm in diameter). Different amounts of GTR particles (70, 80 and 90 wt.%) were used to produce the compounds according to the formulation presented in Table 1. The temperature of the extruder was set at 175°C for all zones to prevent degradation, while the screw speed was set at 100 rpm. As shown in Figure 1, the RHD/EVA was dry-blended and fed to the extruder through the first zone (main feeder), while the GTR particles were fed through a side feeder (zone 4). The overall flow rate was 3 kg/h for all the blends to prevent high motor torque and die pressure associated to the high viscosity of GTR containing compounds. Then, the compounds were cooled in a water bath and pelletized using a model 304 pelletizer (Conair, Stanford, USA). Next, the pellets were dried in a convection oven for 6 h at 75°C to eliminate any residual water before compression molding. The molding was performed at 180°C using 3 min of preheating without pressure and 5 min of pressing under a load of 3 tons using an automatic compression molding press (Carver, AutoFour/1512-PL,H, 3893, USA) with mold dimensions of 115 x 115 x 3 mm^3^.

**Table 1.** Coding and formulation of the samples produced.

Sample code	RHD (wt.%)	NR (wt.%)	RR_1_ (wt.%)	RR_2_ (wt.%)	EVA (wt.%)
N70	30	70	—	—	—
R70	30	—	70	—	—
RR70	30	—	—	70	—
N80	20	80	—	—	—
R80	20	—	80	—	—
RR80	20	—	—	80	—
N90	10	90	—	—	—
R90	10	—	90	—	—
RR90	10	—	—	90	—
N70(5)	25	70	—	—	5
N70(10)	20	70	—	—	10
N70(15)	15	70	—	—	15
N80(5)	15	80	—	—	5
N80(10)	10	80	—	—	10
N80(15)	5	80	—	—	15
R70(5)	25	—	70	—	5
R70(10)	20	—	70	—	10
R70(15)	15	—	70	—	15
R80(5)	15	—	80	—	5
R80(10)	10	—	80	—	10
R80(15)	5	—	80	—	15
RR70(5)	25	—	—	70	5
RR70(10)	20	—	—	70	10
RR70(15)	15	—	—	70	15
RR80(5)	15	—	—	80	5
RR80(10)	10	—	—	80	10
RR80(15)	5	—	—	80	15

### Characterization

#### Contact angle measurements

An optical contact angle analyzer (OCA 15 Plus, Future Digital Scientific Corp., NC, USA) was used at room temperature to measure the contact angle of the materials based on the sessile drop method. Water and ethylene glycol were used as liquids and the average values of five replicates are reported for each sample. The harmonic-mean equation was used to calculate the interfacial tension between each pair of components in the ternary system (RHD, RR and EVA) as follow^30^:

(1) $${\text{γ}}_{\text{ij}}={\text{γ}}_{\text{i}}+{\text{γ}}_{\text{j}}-\frac{4{\text{γ}}_{\text{i}}^{\text{d}}{\text{γ}}_{\text{j}}^{\text{d}}}{{\text{γ}}_{\text{i}}^{\text{d}}+{\text{γ}}_{\text{j}}^{\text{d}}}-\;\frac{4{\text{γ}}_{\text{i}}^{\text{p}}{\text{γ}}_{\text{j}}^{\text{p}}}{{\text{γ}}_{\text{i}}^{\text{p}}+{\text{γ}}_{\text{j}}^{\text{p}}}$$

where ϒ_ij_ is the interfacial tension between components i and j, ϒi is the surface tension of material i and ϒ_i_^p^ and ϒ_i_^d^ are the dispersive and polar contributions of the surface tension of the same material, respectively.

#### Thermogravimetric analysis

Thermal stability of the raw materials was investigated via thermogravimetric analysis (TGA) on a Q5000 IR (TA Instruments, New Castle, USA) at a heating rate of 10°C min^−1^ from 50 to 850°C. The tests were performed in nitrogen and air to evaluate both the thermal and oxidative resistance of the materials.

#### Morphological observation

An Inspect F50 scanning electron microscope (SEM) (FEI, Hillsboro, USA) was used at 15 kV to take micrographs of the GTR particles and observe the interfacial adhesion quality inside the blends. The samples were cryogenically fractured in liquid nitrogen and the surface coated with gold/palladium to be observed at different magnifications.

#### Swelling degree and regeneration degree

The crosslink density was determined according to ASTM D6814 via equilibrium swelling in toluene at room temperature. Firstly, acetone was used to remove the low molecular weight substances of GTR such as processing oils for 16 h. Then, constant weight (0.5 or 1 g) specimens were immersed in toluene at room temperature for 72 h and the swollen samples were weighted. Then samples were dried overnight in an oven at 70°C and the dried samples were weighted. After three repetition for each specimen, the crosslink density was calculated according to the Flory-Rehner equation as^31^:

(2) ve=−[ln(1−vr)+vr+Xvr2][v1(vr13−Vr)/2]

where V_e_ is the crosslink density (mol/cm^3^), V_r_ is the gel volume in the swollen sample, V_1_ is the solvent molar volume (106.2 cm^3^/mol for toluene) and X is the polymer-solvent interaction parameter (0.391).

The gel volume in the swollen sample was calculated according to:

(3) Vr=mrρrmrρr+msρs

where m_r_ and m_s_ are the weight of the dry rubber sample and weight of the solvent absorbed by the sample (g) respectively, while *ρ*_r_ and *ρ*_s_ are the density of the dry rubber sample and density of the solvent (g/cm^3^), respectively.

The regeneration degree of RR_1_ and RR_2_ were evaluated as a function of the crosslink density as:

(4) $$\text{\% Regeneration}=\left(1-\frac{\text{ξ}}{{\text{ξ}}_{0}}\right)\times 100$$

where ξ and ξ_0_ are the crosslink densities of RR (RR_1_ or RR_2_) and NR, respectively.

The sol fraction was determined according to:

(5) $$\text{Sol fraction}\left(\text{\%}\right)=\frac{{\text{W}}_{0}-{\text{W}}_{1}}{{\text{W}}_{0}}\times 100\text{\%}$$

(6) Gel fraction (%)=100−Sol fraction (%)

where W_0_ and W_1_ are the initial weight of the sample (g) and the weight of the dried sample (g), respectively.

In order to analyze the crosslink structure, the swelling degree (swelling ratio) of the blend samples was determined by equilibrium swelling of the specimens in a solvent according to ASTM D471-16a. Around 0.5 g of sample was immersed in 100 mL toluene at room temperature for 72 h to achieve an equilibrium state of swelling. The swollen samples were taken out periodically and excess liquid on the specimen surface was removed with filter paper and the swollen sample was weighed. The swelling degree (Q) was calculated as:

(7) $$\text{Q}=\frac{{\text{W}}_{\text{t}}-{\text{W}}_{\text{i}}}{{\text{W}}_{\text{i}}}\times 100\text{\%}$$

where W_t_ and W_i_ are the weight of the swollen sample (g) and the initial weight of the sample (g), respectively.

#### Mechanical testing

Tensile tests were performed at room temperature according to ASTM D638 using a 500 N load cell and a tensile speed of 10 mm/min on an Instron (Instron, Norwood, USA) universal mechanical tester model 5565. At least 5 dog bone specimens (type V) were used for each blend composition. The average values of the tensile strength (σ_Y_), Young’s modulus (E) and elongation at break (ε_b_) were reported with their standard deviations.

Flexural tests were done on an Instron (Instron, Norwood, USA) model 5565 with a 50 N load cell according to ASTM D790 at room temperature. Rectangular specimens with dimensions of 60 mm x 12.7 mm were tested with 5 repetitions for each sample in a three-point bending mode (span length of 60 mm) at a speed of 2 mm/min.

Notched Charpy impact strength was measured on a Tinius Olsen (Horsham, USA) model 104 at room temperature according to ASTM D256. At least 10 specimens with dimensions of 60 mm x 12.7 mm were used for each sample. Before testing, all the samples were automatically V-notched on a Dynisco (Franklin, USA) model ASN 120m sample notcher 24 h before testing.

#### Physical properties

Hardness values (Shore A and Shore D) were measured by using a 307L model durometer (PTC Instruments, Boston, USA) with 10 measurements for each sample.

Density measurements were performed on a gas (nitrogen) pycnometer Ultrapyc 1200e (Quantachrome Instruments, Boynton Beach, USA). The test was repeated three times for each sample.

## Result and discussion

### TGA analysis

**Figure 2.**
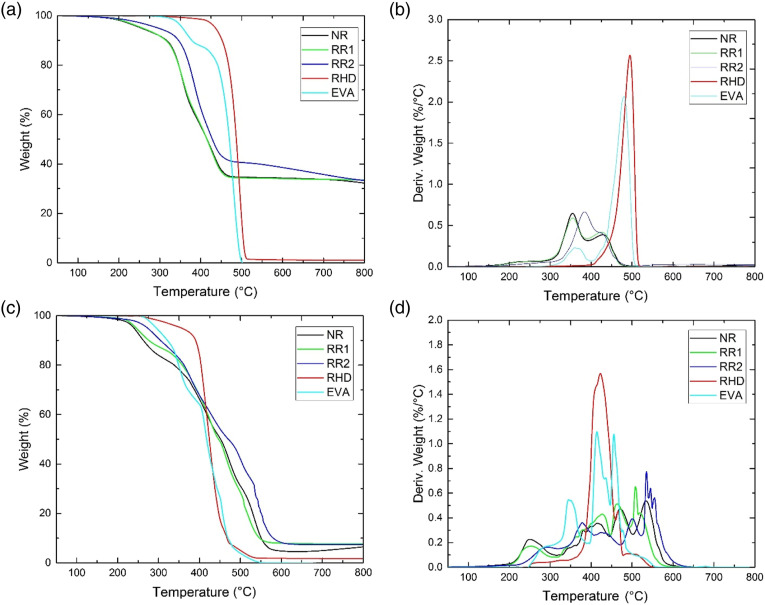
TGA and DTG curves of the raw materials in: (a,b) nitrogen and (c,d) air.

Figure 2 presents the weight curves (TGA) and their derivative (DTG) for the raw materials used: RHD, EVA and GTR (NR, RR_1_ and RR_2_). The curves under nitrogen show that the initial degradation of RHD is around 400°C, while the maximum decomposition rate is around 490°C. The decomposition of EVA occurs in two steps. The initial weight loss between 250 and 350°C is related to the deacetylation process in which acetic acid is released and the formation of C=C bonds along the polymer backbone. The second degradation step (between 350 and 500°C) is attributed to the oxidation and volatilization of hydrocarbons resulting from the decomposition of the backbone.^32^ The thermal decomposition of all GTR (NR, RR_1_ and RR_2_) obtained from same source of OTR recycled rubber begins around the same temperature (200°C), so the processing temperature for all TPE blends should not exceed 200°C to avoid negative effects on the final TPE properties. The first decomposition step between 200 and 350°C can be related to the evaporation or decomposition of processing oils, additives and other compounds with low molar mass and/or low boiling temperature in the GTR formulation.^33^ The second decomposition step between 350 and 430°C can be related to the decomposition of the polymeric material present in the tire rubber such as natural rubber. The last stage of decomposition (430 to 800°C) is ascribed to the residual materials, mainly the inorganics. The char residues in an inert atmosphere (nitrogen) are about 32.2, 33.6 and 33.9% for NR, RR_1_ and RR_2_ respectively, indicating the presence of inorganic particles in GTR. The breakup of crosslinks during regeneration can thermally destabilize the rubber and promote its degradation at lower temperatures. However, the higher char residue of RR (RR_1_ and RR_2_) particles than NR particles can be attributed to the presence of higher amounts of carbon black which act as a physical barrier and adsorb low molecular weight (MW) volatile products formed during thermal degradation, thus improving their apparent thermal stability as reported elsewhere.^34^ The DTG curve of NR, RR_1_ and RR_2_ in nitrogen shows a wide bump between 350 and 430°C which is composed of two peaks. This two-stage decomposition of GTR under an inert atmosphere might be attributed to the different decomposition temperature of natural and synthetic rubbers.^35^ The small peak or shoulder close to the main peaks around 220°C is representative of other additives degradation.^17^

It was expected that exposure to oxygen under air decreases the thermal stability of all recycled materials. RHD has almost no branches in its molecular structure (HDPE) leading to high thermal stability. As expected, the thermal stability of RHD decreased due to the presence of oxygen in air, while the initial weight loss of RHD started earlier at around 300°C (Figure 2(c)) and the DTG curve of RHD shows a single peak at 430°C.^36^ The TGA curves of EVA under air shows that the thermal degradation included similar stages as the TGA curves in N_2_. The loss of acetic acid, because of the decomposition of vinyl acetate groups and the decomposition of polyethylene chains, is responsible for this degradation stage under air, while almost no residue was left.^37^Figure 2(c) shows that the initial weight loss of GTR (NR, RR_1_ and RR_2_) starts around 200°C and all GTR particles degraded almost completely around 570°C. Also, the DTG curves of GTR present several peaks between 250 and 530°C related to the GTR decomposition and its complex structure as a mixture of various components. The highest decomposition temperature of GTR might be attributed to the oxidation of carbon residues generated at lower temperatures, leading to the formation of carbon dioxide.^38^

### Swelling properties

Table 2 presents the sol fraction, gel fraction, crosslink density and regeneration degree of the GTR particles before (NR) and after regeneration (RR_1_ and RR_2_). This information is a direct quantification of the extent of rubber network breakup during regeneration. Upon GTR regeneration, the sol fraction increased from 2.6% for NR, to 6.5% and 11.6% for RR_1_ and RR_2_, respectively. Higher sol fraction of RR particles compared to NR particles is related to random crosslink (polysulfidic, disulfidic and monosulfidic) and polymer chain scission by mechanical shearing and heat during the thermo-mechano-chemical regeneration of GTR (NR). The shear forces might cause unselective scission of the rubber main chains and the reduction of the performance of RR particles.^39^ The crosslink density of NR particles decreased from 7.2×10^−4^ mol/cm^3^ to 6.1×10^−4^ mol/cm^3^ (RR_1_) and 5.5×10^−4^ mol/cm^3^ (RR_2_) after rubber regeneration. The immobilized fraction of rubber chains decreased with decreasing crosslink density of RR because of the vulcanized network is partially broken-up which increased the chain mobility and flexibility of the polymer, while lower chain restriction leads to low rigidity and modulus after regeneration. It is clear that both the gel fraction and crosslink density of NR decreased after regeneration, so the breakdown of the rubber crosslinked structure led to restore GTR plasticity and reprocessability. The regeneration degree of RR_1_ and RR_2_ are 15.2% and 24.1%, respectively. Lower regeneration degree of RR_1_ means that a higher crosslink density remains in the particles compared to RR_2_. But more crosslinked molecular chains will result in a more uneven stress distribution and lower tensile strength as described later.^40^ Another reason for the higher regeneration degree of RR_2_ might be related to a more selective cleavage of the sulfur crosslinks with less molecular main chains scission compared to the RR_1_ regeneration in which main chain scission is dominant resulting in a drop of MW and a loss of mechanical strength for RR_1_ blends.^41^ The regeneration degree is the main parameter controlling the processing and mechanical performance of TPE blends filled with RR particles as described later.

**Table 2.** Sol and gel fraction, crosslink density and regeneration degree of the GTR particles.

Sample	**Sol fraction (%)**	**Gel fraction (%)**	**Crosslink density (10** ^**−4**^ ** mol/cm** ^**3**^ **)**	Regeneration degree (%)
**NR**	2.6 (0.1)^a^	97.4 (0.1)	7.2	—
**RR**_**1**_	6.5 (0.1)	93.5 (0.1)	6.1	15.2 (0.6)
**RR**_**2**_	11.6 (0.2)	88.4 (0.2)	5.5	24.1 (0.9)

^a^(Standard deviation).

The swelling degree of rubber compounds represents the sorption behavior of a solvent, such as toluene, to determine the crosslink density of TPE. An inverse relation between the swelling degree and the crosslink density exists as lower swelling degree implies a higher crosslink density.^42^ The swelling test was performed to evaluate the swelling degree of the TPE blends to determine the effect of GTR regeneration and toluene uptake on the mechanical properties as discussed later. The swelling degree of compatibilized (RHD/GTR/EVA) and uncompatibilized (RHD/GTR) blends are presented in Table 3. Increasing the GTR content leads to higher swelling degrees due to a higher elastomer content, which in turn results in the absorption of more toluene. This observation is in agreement with other reports showing that GTR particles contain some soluble molecules which can absorb the solvent and swell.^43^ Increasing the GTR content by 10% (from 70 to 80 wt.%) in RHD/GTR blends increased the swelling degree of N80 from 138 to 145%, while the swelling degree of RR_1_ and RR_2_ blends increased from 160% to 169% and from 172% to 180%, respectively. This difference is attributed to the lower resistance to non-polar solvents of partially destroyed crosslinked structure of RR particles which promotes swelling.^44^ The higher swelling degree of RR blends compared to NR blends is associated to the lower crosslink density of RR_1_ (6.1×10^−4^ mol/cm^3^) and RR_2_ (5.5×10^−4^ mol/cm^3^) compared to NR (7.2×10^−4^ mol/cm^3^) particles ^12^ Also, the presence of fillers and other non-crosslinkable products in the blends can affect the swelling degree, as the RHD/GTR/EVA blends show lower toluene uptake compared to RHD/GTR blends. For example, by adding 10 wt.% EVA, the swelling degree of RHD/GTR (20/80) blends decreased by 1.4% (from 145 to 143%), 3.9% (from 169 to 163%) and 2.9% (from 180 to 175%) for NR, RR_1_ and RR_2_ blends, respectively. This observation might be related to good filler/matrix interaction favorable for interfacial interaction resulting in lower voids in the blends leading to more difficult solvent penetration.^45^

**Table 3.** Swelling degree of the RHD/GTR and RHD/GTR/EVA blends. See Table 1 for sample composition.

Sample	Swelling degree (%)	Sample	Swelling degree (%)	Sample	Swelling degree (%)
N70	138 (0.7)	**R70**	160 (0.8)	**RR70**	172 (0.9)
N80	145 (0.6)	**R80**	169 (0.8)	**RR80**	180 (0.8)
N90	154 (0.8)	**R90**	187 (0.8)	**RR90**	192 (0.7)
N70(5)	135 (0.4)	**R70(5)**	159 (0.6)	**RR70(5)**	168 (0.5)
N70(10)	131 (0.3)	**R70(10)**	157 (0.4)	**RR70(10)**	164 (0.5)
N70(15)	132 (0.8)	**R70(15)**	143 (0.7)	**RR70(15)**	154 (0.7)
N80(5)	144 (0.5)	**R80(5)**	168 (0.4)	**RR80(5)**	178 (0.6)
N80(10)	143 (0.6)	**R80(10)**	163 (0.8)	**RR80(10)**	175 (0.9)
N80(15)	140 (0.3)	**R80(15)**	162 (0.4)	**RR80(15)**	1 (0.4)

### Morphological observation

**Figure 3.**
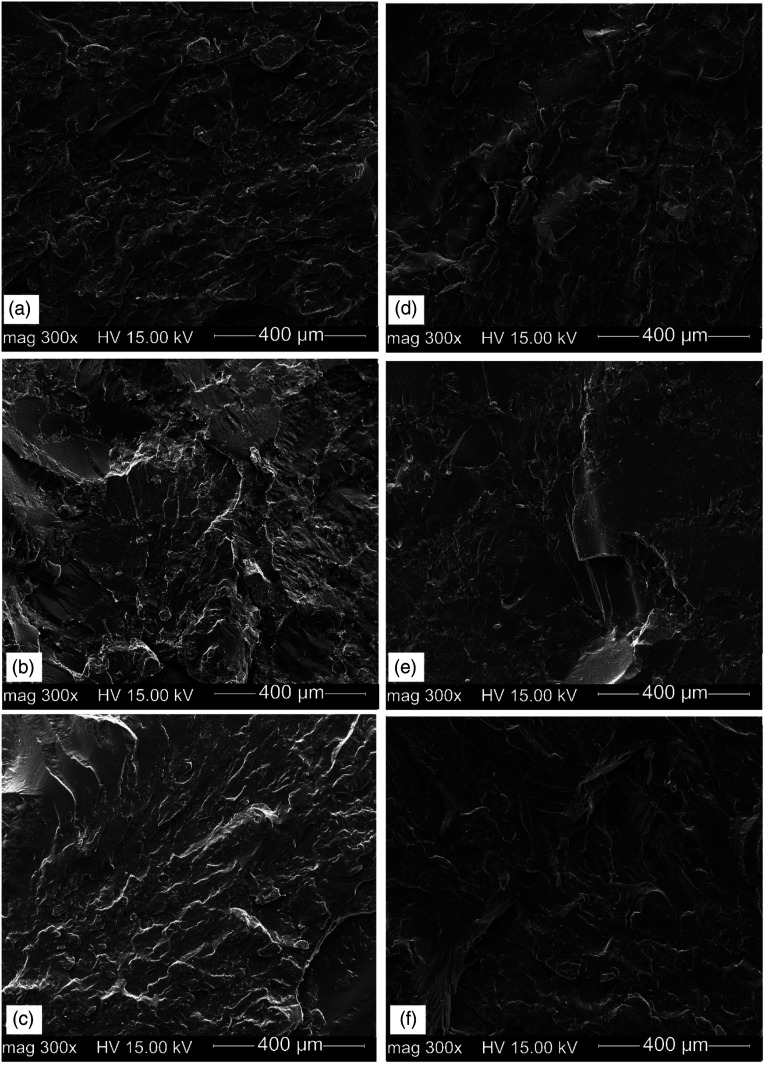
SEM micrograph of: (a) N80, (b) R80, (c) RR80, (d) N90, (e) R(90) and (f) RR(90). See Table 1 for sample composition.

Figure 3 presents SEM micrographs of the RHD/GTR blends at two different GTR content (80 and 90 wt.%) to show the effect of the blend composition and regeneration process on the GTR distribution and the interfacial adhesion between the components. Increasing the GTR concentration is expected to create a less homogeneous structure due to the difficult dispersion of highly crosslinked rubber particles in a highly viscous thermoplastic matrix.^13^ As shown in Figure 3, a clear distinction between the rubber particles (NR, RR_1_ and RR_2_) and RHD with interfacial gaps implies a weak interface which is getting worse with increasing the GTR content from 80 to 90 wt.% (Figure 3(d)–(f)). In fact, the clean fractured surface of the blends indicates an easy removal of weekly connected GTR particles from the RHD thermoplastic related to a lack of strong interfacial bonding which is more evident in RR_1_ blends (Figure 3(b) and (e)) and higher GTR loading (90 wt.%). The high surface energy between the GTR and RHD leads to limited interfacial stress transfer, so failure occurs at the interface as crack initiation and propagation are easy.^7^ Melt mixing of RHD with 80 wt.% RR_2_ led to the production of more homogeneous blends (Figure 3(c)) compared to the blends containing the same concentration of NR (Figure 3(a)) or RR_1_ (Figure 3(b)). The higher sol fraction of RR_2_ (11.6%) results in better bonding with the polymer matrix to improve interfacial adhesion between the phases.^46^

**Figure 4.**
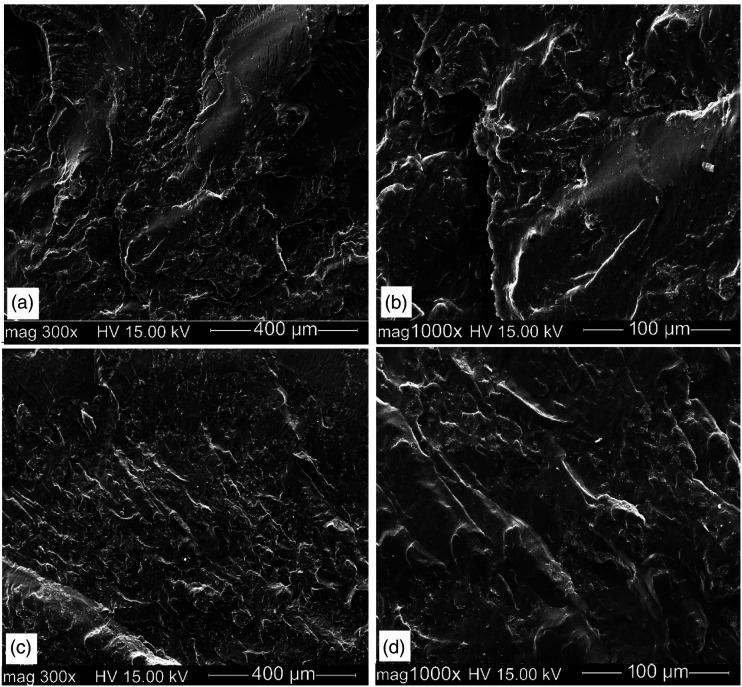
SEM micrographs of: (a,b) R80(10) and (c,d) RR80(10).

Figure 4 shows typical SEM micrographs of the fractured surface of RHD/GTR/EVA blends containing 10 wt.% EVA to determine the effect of a compatibilizer on the state of interfacial adhesion and compatibility in the blends. It is known that in multicomponent blends, the fracture behavior strongly depends on the interfacial bonding between the components.^7^ So good compatibility in the blends leads to failure starting in the continuous phase instead of the interface generating higher mechanical properties.^13^

The clean and smooth fractured surface of R80(10) (Figure 4(a)) implies an easy detachment and pull-out of RR_1_ particles from the RHD matrix under tensile stress. On the other hand, the rough fracture surface of RR80(10) (Figure 4(c)) indicates that RR_2_ particles are strongly embedded in the RHD matrix and higher energy is required for their detachment. This observation is related to the incomplete dispersion and agglomeration of RR_1_ particles due to the low compatibility between the recycled rubber (RR_1_) and RHD and low stress transfer.^26^ In RR80(10), the rubber particles are more uniformly dispersed in RHD (Figure 4(c)) and detection of the rubber phase is difficult even at high magnification (Figure 4(d)). Li et al.^26^ calculated the interfacial tension between polymer pairs in HDPE/GTR/elastomer composites and predicted that the lower interfacial tension of GTR/elastomer compared to HDPE/GTR can lead to GTR encapsulation by the elastomer (EVA). Also, Lima et al.^11^ observed that EPDM was able to encapsulate GTR particles to create an interface between a thermoplastic PP matrix and crosslinked GTR particles, thus improving the blends compatibility. As shown in Figure 4(d), no gap between each phase is observed so it can be concluded that some rubber particles are covered by the elastomer leading to a lower surface energy between the RR_2_ and RHD phases improving the fine dispersion of RR_2_ particles. Similar observations have been reported for PP/filler/elastomer composites with the addition of EVA as a polar elastomer covering filler particles (calcium carbonate) and resulting in PP/filler composites with an encapsulated structure.^47^

**Figure 5.**
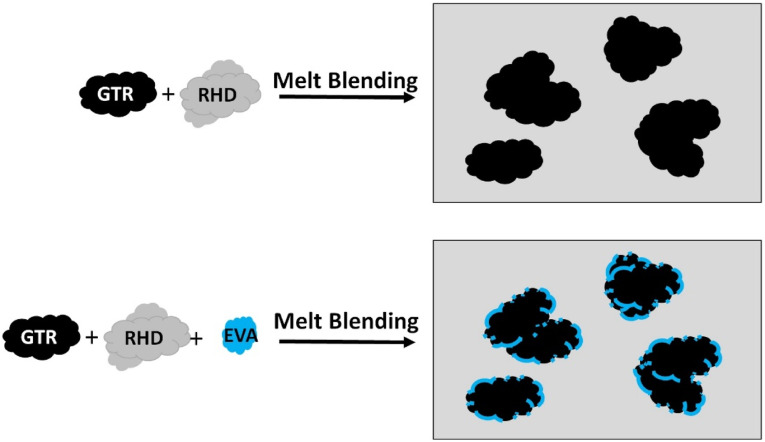
Proposed compatibilization mechanism of a thermoplastic (recycled high-density polyethylene)/ground tire rubber/elastomer (ethylene vinyl acetate).

To predict the morphology of immiscible ternary blends, the spreading coefficient thermodynamic model can be used. For two discrete minor phases (polymer j and k) dispersed in a matrix phase (polymer i) the following equation can be used^48^:

(8) $${\text{λ}}_{\text{ijk}}={\text{γ}}_{\text{ik}}-{\text{γ}}_{\text{ij}}-{\text{γ}}_{\text{jk}}$$

where λ is the spreading coefficient and ϒ is the interfacial tension between the components of the ternary system which can be measured by surface tension values based on contact angles measurements and the harmonic-mean equation (equation (1)). As shown in Table 4, high interfacial tension between the thermoplastic matrix and rubber particles indicates that the binary TPE are immiscible. The lower interfacial tension of EVA/RR_2_ (2.3 mN/m) compared to that of RHD/RR_2_ (5.4 mN/m) and RHD/EVA (2.8 mN/m) suggests that the phase morphology of this multicomponent polymer system should correspond to the lowest free energy when the rubber phase is encapsulated by the elastomer copolymer in the RHD matrix. Figure 5 presents a schematic representation of the compatibilization mechanism for a thermoplastic/rubber/elastomer system in which the recycled rubber particles are encapsulated by EVA. The spreading coefficients for the ternary RHD/RR_2_/EVA system (λ_jki_= 0.3) predicts partial wetting and a tendency of the elastomer phase (EVA) to spread at the interface of RHD and RR resulting rubber encapsulation by EVA in agreement with similar observation.^26^ It is expected that decreasing the rubber particles size results in higher probability of rubber particles encapsulation by the elastomer leading to the formation of a strong interface between the components.^6^ However, the presence of processing oil in RR_1_ may lead to particle swelling and agglomeration, so large rubber particles with lower specific surface area limit the possibility of RR_1_ encapsulation by EVA to form a strong interphase.

**Table 4.** Interfacial tension between the components and spreading coefficients.

Polymer pairs	Interfacial tension, ϒ (mN/m) at 25°C	Spreading coefficient (λ) RHD/RR_2_/EVA (i/j/k)
RHD/RR_2_	5.4	λ_jik_ −5.9
RHD/EVA	2.8	λ_jki_ 0.3
RR_2_/EVA	2.3	λ_ijk_ −4.9

i = RHD, j = RR_2_, and k = EVA

### Mechanical properties

#### Tensile properties

**Figure 6.**
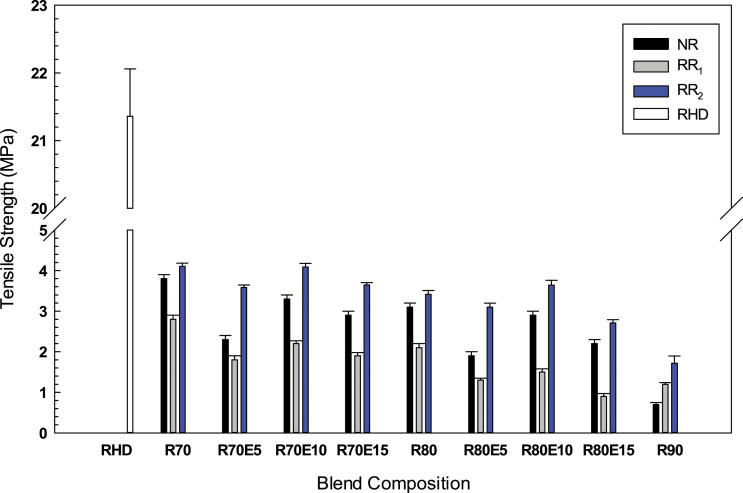
Tensile strength of the recycled high-density polyethylene/ground tire rubber and recycled high-density polyethylene/ground tire rubber/ethylene vinyl acetate compounds blends. See Table 1 for sample composition.

As shown in Figure 6, the tensile strength of RHD decreased from 21.3 MPa to 3.1, 2.1 and 3.4 MPa after melt blending with 80 wt.% of NR, RR_1_ and RR_2_, respectively. This can be attributed to the presence of GTR particles acting as stress concentration points (crack initiation points)^14^ and lack of entanglement between the crosslinked GTR and thermoplastic matrix resulting in low affinity (incompatibility) and weak interfacial adhesion.^25^ Other reasons for the lower tensile properties after rubber incorporation originates from GTR exposure to ozone, mechanical and thermal degradation during its service life and also during the grinding process. The tensile strength of RR_2_ blends is higher than for RR_1_ blends because of the lower gel fraction (88.4%) and crosslink density (5.5×10^−4^ mol/cm^3^) of RR_2_ compared to the gel fraction (93.5%) and crosslink density (6.1×10^−4^ mol/cm^3^) of RR_1_. Higher crosslink density and limited mobility of the RR_1_ molecular chains caused uneven stress distribution and the lower tensile strength of RR_1_ blends.^26^ Addition of EVA (10 wt.%) showed slight tensile strength increase from 3.4 MPa for RR80 to 3.6 MPa for RR80(10) because of enhanced dispersion of recycled rubber particles in the thermoplastic resin (compatibilizing effect of EVA) and enhanced interaction between RHD and the soluble fraction of RR_2_ (11.6%). However, incorporation of 15 wt.% EVA led to a tensile strength drop since an excessive amount of compatibilizer partially destroys the continuity of the RHD matrix leading to lower mechanical strength.^49^

**Figure 7.**
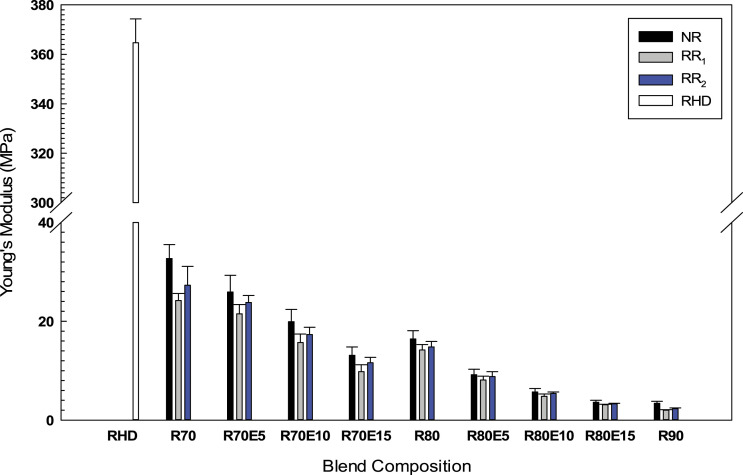
Young’s modulus of the RHD/GTR and RHD/GTR/EVA blends. See Table 1 for sample composition. RHD: recycled high-density polyethylene; GTR: ground tire rubber; EVA: ethylene vinyl acetate.

As shown in Figure 7, the Young’s modulus of the RHD/GTR composites decreased from 364.7 MPa (RHD) to 16.4, 14.2 and 14.8 MPa after melt blending with 80 wt.% NR, RR_1_ and RR_2_, respectively. Significant decrease in Young’s modulus with GTR content was expected due to the inherent soft nature of the rubber phase.^5^ Incorporation of a compatibilizer (EVA) led to better compatibility between RHD and GTR improving the stress transfer from the matrix to the rubber particles leading to lower rigidity of the compounds.^50^ Addition of 10 wt.% EVA into RHD/GTR (20/80) decreased the Young’s modulus of NR, RR_1_ and RR_2_ blends by 65% (from 16.4 to 5.7 MPa), 66% (from 14.2 to 4.8 MPa) and 63% (from 14.8 to 5.4 MPa), respectively. Also, a decreasing trend in Young’s modulus with increasing EVA content is ascribed to the softening or dilution effect of the soft elastomeric phase (EVA) with low modulus (26 MPa) compared to RHD (364 MPa). Similar reports on ternary blends containing an elastomer phase showed lower modulus than those of binary TPE blends based on a polymer matrix and recycled rubber.^7,20^ Mészáros et al.^7^ also concluded that increasing the EVA content (from 10 to 30 wt.%) substantially decreased the Young’s modulus of rLDPE/LDPE/GTR/EVA (40/20/30/10) from 310 MPa to 180 MPa (40/0/30/30).

**Figure 8.**
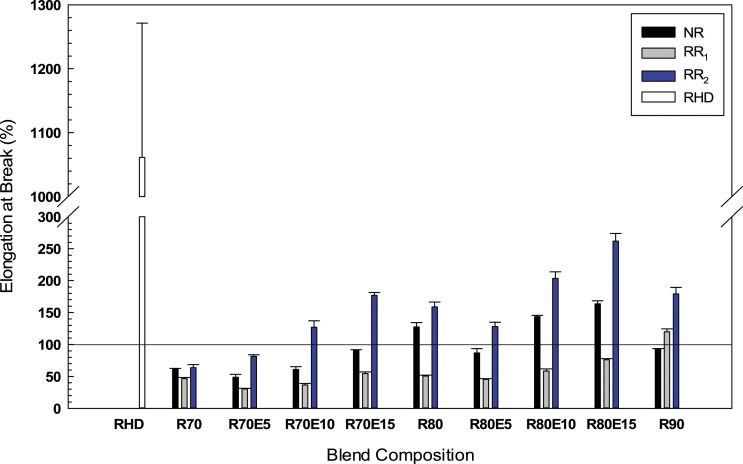
Elongation at break of the RHD/GTR and RHD/GTR/EVA blends. See Table 1 for sample composition. RHD: recycled high-density polyethylene; GTR: ground tire rubber; EVA: ethylene vinyl acetate.

The elongation at break is the most important property to determine the compatibility and homogeneity of TPE blends.^13^ Although the elongation at break of NR blends increased from 61.9 to 127.6% with increasing NR content from 70 to 80 wt.% due to the presence of a more elastic content, the values are much less than for the RHD matrix (1060%) (Figure 8). Similarly, Li et al.^26^ reported decreasing elongation at break of HDPE from 800 to 33% after the addition of 40 wt.% GTR. The poor GTR distribution in the polymer matrix promoted particle-particle interactions and contributed to weak sites upon stress-transfer between the rubber and matrix interface which are failure points.^23^ In general, the regeneration process leads to more free chains via partial breakdown of the rubber network improving possible interactions between GTR and the corresponding polymer matrix. However, melt blending RHD with 80 wt.% regenerated rubber show different elongation at break of 50% and 159% for R80 and RR80, respectively. This observation can be attributed to the higher sol fraction of RR_2_ (11.6%) compared to RR_1_ (6.5%) which promoted interfacial adhesion between the soluble content of RR_2_ and RHD and hence higher plastic deformation of RR80.^51^ Also, it can be proposed that a more efficient regeneration of RR_2_ (24.1%) to break-up the vulcanized structure with less scission of the main chains contributes to the higher plastic deformation of RR_2_ blends.^52^ The regeneration process is not a 100% selective rupture of sulfur bonds alone and might also produce degradation of the main chains of the recycled rubber during regeneration (extensive shear and high temperature) lowering the MW and degrading the tensile properties.^40^

Figure 8 shows that the addition of 10 wt.% EVA increased the elongation at break of N80(10), R80(10) and RR80(10) to 144% (from 128%), 58% (from 51%) and 203% (from 159%), respectively. In agreement with other observations, this behavior shows the rubber-toughening effect and enhanced interfacial adhesion caused by the presence of an elastomer. Incorporation of EVA decreases the stress concentration around the particles (GTR encapsulation) and inhibits fracture phenomena.^6,7,26^ It must be pointed out that increasing the EVA content also decreased the RHD content in our case.

**Figure 9.**
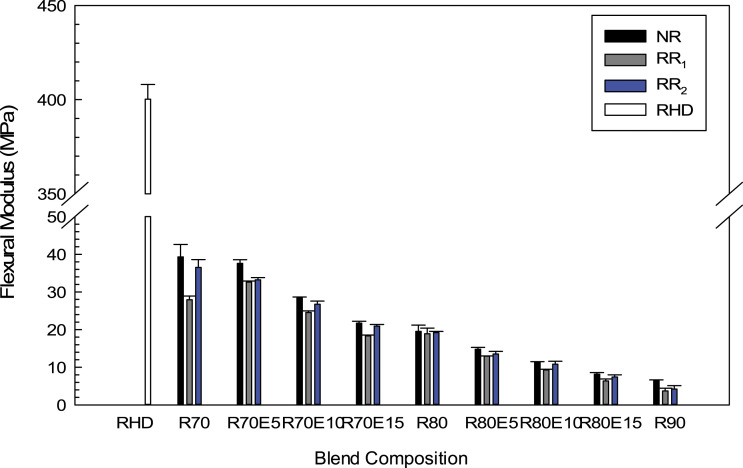
Flexural modulus of the RHD/GTR and RHD/GTR/EVA blends. See Table 1 for sample composition. RHD: recycled high-density polyethylene; GTR: ground tire rubber; EVA: ethylene vinyl acetate.

The results of flexural modulus are presented in Figure 9. Increasing the GTR content from 70 to 80 wt.% decreased the flexural modulus of NR, RR_1_ and RR_2_ blends by 50% (from 39.3 to 19.5 MPa), 32% (from 27.9 to 18.8 MPa) and 47% (from 36.5 to 19.2 MPa), respectively. The soft nature of GTR as a low modulus phase and the presence of interfacial voids/defects are responsible for the decreasing flexural modulus trend with increasing GTR content similar to the Young’s modulus (Figure 7). Incorporation of NR with a vulcanized structure and higher crosslink density than RR_1_ and RR_2_ particles (Table 2), as well as further chain mobility restriction, led to more rigidity and higher flexural modulus of the NR blends. It should be noticed that GTR regeneration leads to smaller fragment and shorter chains of RR which can act as plasticizers, as well as the presence of a processing oil used in regeneration, so the flexural modulus of blends with RR_1_ and RR_2_ are lower than that of NR blends. The flexural modulus also substantially decreased with the presence of a with low modulus EVA. For example, adding 10 wt.% of EVA into blends with 80 wt.% GTR decreased the flexural modulus by 42% (from 19.5 to 11.3 MPa), 50% (from 18.8 to 9.3) and 43% (from 19.2 to 10.8 MPa), for NR, RR_1_ and RR_2_ blends, respectively.

#### Impact strength

**Figure 10.**
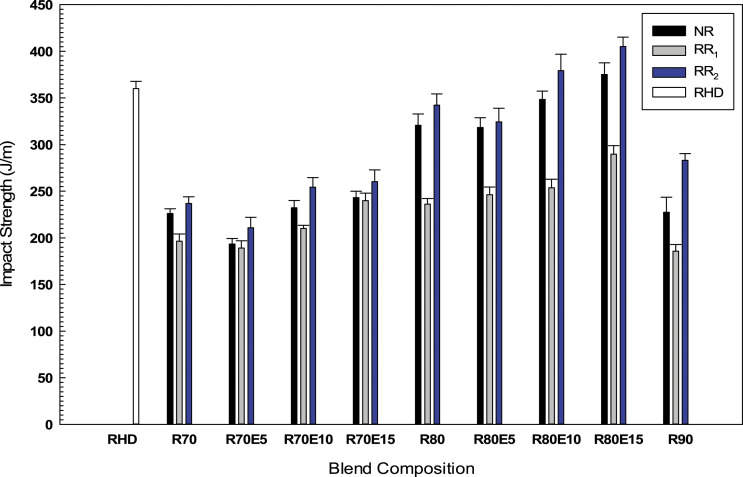
Impact strength of the RHD/GTR and RHD/GTR/EVA blends. See Table 1 for sample composition. RHD: recycled high-density polyethylene; GTR: ground tire rubber; EVA: ethylene vinyl acetate.

As shown in Figure 10, increasing the GTR content from 70 to 80 wt.% increased the impact strength for all the formulations. The higher impact strength of RR80 (342 J/m) compared to that of N80 (321 J/m) and R80 (236 J/m) can be attributed to better interfacial adhesion between RR_2_ and RHD (Figure 3–4) inhibiting crack propagation. However, further GTR increase (up to 90 wt.%) led to a drop in impact strength because of poor sample homogeneity (Figure 3).^26^ This indicates that a GTR concentration between 80 and 90 wt.% seems to be a critical point for these compounds. The addition of 10 wt.% EVA into the blends containing 80 wt.% GTR increased the impact strength of NR, RR_1_ and RR_2_ blends by 9% (from 321 to 348 J/m), 7% (from 236 to 254 J/m) and 11% (from 342 to 379 J/m), respectively. The presence of EVA improves the toughness and increases the absorbed energy before crack initiation and propagation by inducing interfacial bonding in RHD/GTR/elastomer blends. EVA can promote a more uniform GTR dispersion in the matrix by encapsulating the GTR particles and decreasing the surface energy leading to better RHD deformability around the GTR particles.^6,26^

#### Hardness

**Figure 11.**
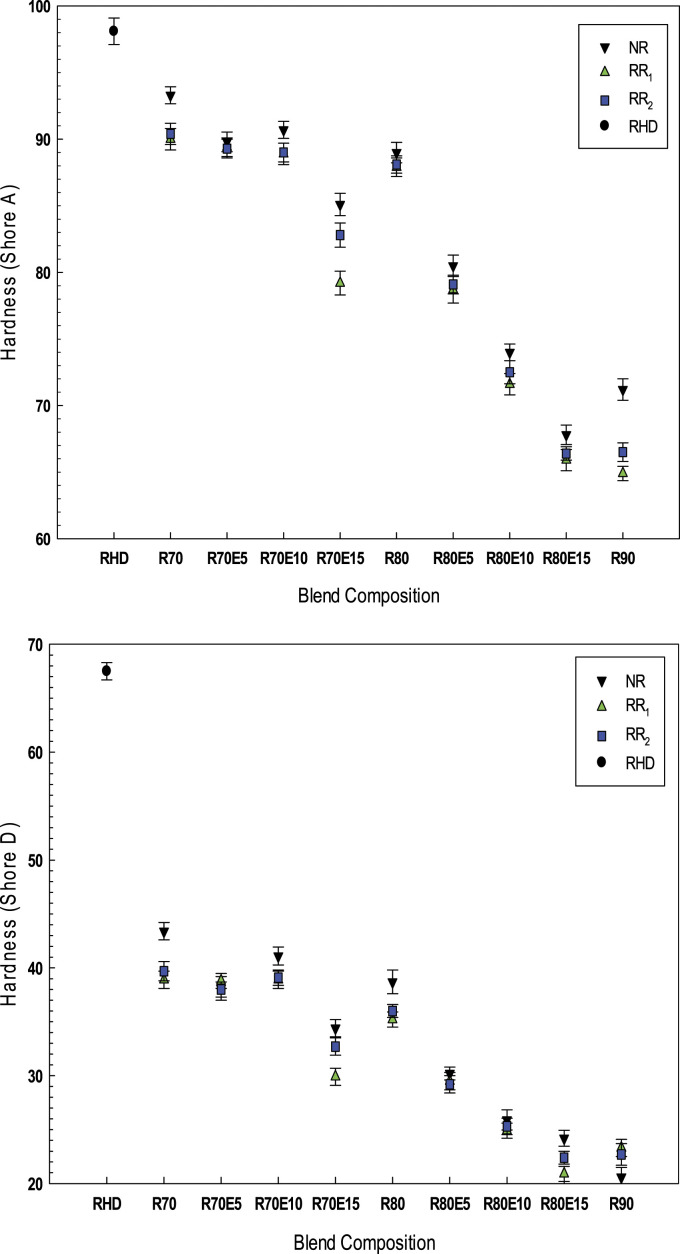
Hardness (Shore A and Shore D) of the RHD/GTR and RHD/GTR/EVA blends. See Table 1 for sample composition.

Figure 11 presents the hardness (Shore A and Shore D) of the blends as a function of different GTR types (NR, RR_1_ and RR_2_) for compatibilized and uncompatibilized samples. In general, the hardness of TPE is influenced by the elastic modulus and crosslink density. Despite the presence of carbon black in GTR, adding recycled rubber particles as an elastomeric component into a rigid (thermoplastic) phase results in lower hardness values.^13^ For instance, melt blending of 80 wt.% GTR with RHD decreased the Shore A hardness of RHD from 98 to 89 for NR blends, while the value are 87 and 88 for R80 and RR80, respectively. Also, the Shore D values decreased from 67 for RHD to 38, 35 and 36 after melt blending with 80 wt.% NR, RR_1_ and RR_2_, respectively. The hardness results can also be used as a rough approximation of the crosslink level of the blends. The regeneration process led to lower rigidity of the blends due to improved chain mobility (lower crosslink density) and the presence of processing oil in the recycled rubber as reported by Shaker and Rodrigue.^5^ The hardness of R80(10) decreased by 16 points Shore A (87 to 71) and 11 points Shore D (35 to 24), while the hardness of RR80(10) decreased by 15 points Shore A (88 to 73) and 10 points Shore D (36 to 26). Significant decrease in hardness for the compatibilized blends is related to the presence of a soft compatibilizer (10 wt.% EVA) which promoted elasticity and softness.^50^

#### Density

**Figure 12.**
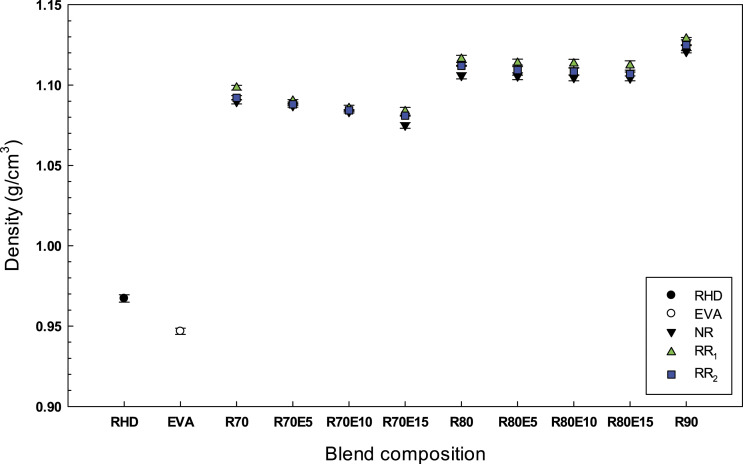
Density of RHD/GTR and RHD/GTR/EVA blends. See Table 1 for sample composition. RHD: recycled high-density polyethylene; GTR: ground tire rubber; EVA: ethylene vinyl acetate.

Figure 12 presents the density of the blends as a function of GTR content for compatibilized and uncompatibilized blends. In general, the density increased with GTR content due to its higher density (NR = 1.169 g/cm^3^, RR_1_ = 1.246 g/cm^3^ and RR_2_ = 1.193 g/cm^3^) compared to RHD (0.967 g/cm^3^). Higher density of the blends containing RR_1_ can be related to the presence of a processing oil in RR_1_ particles resulting in slightly higher (about 1%) density of all RR_1_ blends. The density of the compatibilized samples is slightly lower (1%) than the uncompatibilized compounds since EVA has the lowest density (0.946 g/cm^3^). Once again, increasing the EVA content decreases the RHD content.

## Conclusion

GTR regeneration was proposed as a promising approach to improve the interfacial interaction between the soluble fraction of recycled rubber (RR) and a recycled thermoplastic matrix (high-density polyethylene, RHD). The samples were produced via continuous melt-mixing of RHD with different types of ground tire rubber GTR (NR, RR_1_ and RR_2_) in the range of 70 to 90 wt.% using a twin-screw extruder. Also, recycled EVA was used as a polar compatibility/interfacial adhesion promoter to produce ternary blends of RHD/GTR/EVA with different EVA content (5–15 wt.%).

The results confirmed the influence of the GTR regeneration and concentration on the mechanical and morphological properties of the resulting TPE. The morphological and mechanical results revealed that an efficient breakdown of the crosslinked network of RR_2_ (regeneration degree of 24.1%) with a low gel fraction (88.4%) and crosslink density (5.5×10^−4^ mol/cm^3^) would contribute to sufficient chain entanglement between RR_2_ and RHD to create a strong interface leading to higher plastic deformation and toughness. But partial substitution of RHD by EVA (5, 10 and 15 wt.%) gave rise to higher TPE homogeneity and compatibility. For example, RHD/GTR/EVA (10/80/10) blends showed lower toluene uptake compared to RHD/GTR (80/20) blends since the addition of 10 wt.% EVA slightly decreased the swelling ratio of N80(10), R80(10) and RR80(10) between 1.4% and 3.9%. It can be concluded that good filler/matrix interaction resulted in lower voids and less solvent penetration into the compatibilized blends. For the mechanical properties, the presence of 10 wt.% EVA increased the elongation at break of RR80(10) to 203% (from 159% without EVA), while the elongation at break of N80(10) and R80(10) increased to 144% (from 128%) and to 75% (from 51%), respectively. Lower plastic deformation of RR_1_ blends compared to that of RR_2_ and even NR blends might be attributed to the regeneration process (extensive shear and high temperature) which caused partial degradation of the main rubber chain instead of a selective rupture of the sulfur crosslinks. Also, EVA addition in N80(10), R80(10) and RR80(10) increased the impact strength between 9% and 11%.

It can be concluded that EVA addition can promote a more uniform GTR dispersion (especially RR_2_) by particles encapsulation to create a strong interface and increase the deformation ability of the RHD matrix around these particles to improve plastic deformation and toughness.
